# Primary and Acquired Resistance against Immune Check Inhibitors in Non-Small Cell Lung Cancer

**DOI:** 10.3390/cancers14143294

**Published:** 2022-07-06

**Authors:** Qinying Sun, Xiangzhen Wei, Zhonglin Wang, Yan Zhu, Weiying Zhao, Yuchao Dong

**Affiliations:** 1 Department of Respiratory and Critical Care Medicine, Changhai Hospital, The First Affiliated Hospital of Naval Medical University (Second Military Medical University), Shanghai 200001, China; sqy20031@163.com; 2 Medical College, Shaoxing University, Shaoxing 311201, China; xxw736@case.edu (X.W.); zhonglw4@uci.edu (Z.W.); yan.zhu@uth.tmc.edu (Y.Z.)

**Keywords:** non-small cell lung cancer, immune check inhibitors, primary and acquired resistance

## Abstract

**Simple Summary:**

NSCLC accounts for approximately 84% of lung malignancies and the clinical application of ICIs provides a novel and promising strategy. However, approximately 80% of NSCLC patients do not benefit from ICIs due to drug resistance complicated by disciplines and diverse mechanisms. Through this review, we provide a whole map of current understanding of primary and acquired resistance mechanisms in NSCLC. In the first part, resistance mechanisms of 6 FDA-approved ICIs-related primary resistance are collected and arranged into 7 steps of the well-known cancer-immunity cycle. Acquired resistance induced by ICIs are summarized in the second part. In the third part, we discuss the future direction, including the deeper understanding of tumor microenvironment and the combinational treatment. Through this review, clinicians can get clear and direct clues to find the underlying mechanisms in patients and translational researchers can acquire several directions to overcome resistance and apply new combinational treatment.

**Abstract:**

Immune checkpoint inhibitors have emerged as the treatment landscape of advanced non-small cell lung cancer (NSCLC) in recent years. However, approximately 80% of NSCLC patients do not benefit from ICIs due to primary resistance (no initial response) or acquired resistance (tumor relapse after an initial response). In this review, we highlight the mechanisms of primary and secondary resistance. Furthermore, we provide a future direction of the potential predictive biomarkers and the tumor microenvironmental landscape and suggest treatment strategies to overcome these mechanisms.

## 1. Introduction

Non-small cell lung cancer (NSCLC) accounts for approximately 84% of lung malignancies and main subtypes include adenocarcinoma, squamous cell carcinoma, and large cell carcinoma [1]. As a severe global health challenge, NSCLC caused over 150,000 deaths in 2020 [2]. Due to late diagnosis, limited treatment strategies, and drug resistance, 5-year overall survival (OS) for NSCLC is approximately 24% and the OS for NSCLC with distant metastasis is merely 5.5% [3]. Recently, the clinical application of immune checkpoint inhibitors (ICIs) has provided a novel and promising strategy. Currently, the US Food and Drug Administration (FDA) approves three categories of ICIs for the treatment of NSCLC, including cytotoxic T lymphocyte antigen 4 (CTLA-4) inhibitors, programmed death 1 receptor (PD-1) inhibitors, and programmed cell death receptor ligand 1 (PD-L1) inhibitors. In total, 6 ICIs have been approved for clinical use against NSCLC, including ipilimumab (a fully human IgG1 antibody targeting CTLA-4), pembrolizumab (a humanized, IgG4 antibody targeting PD-1), nivolumab (a fully human IgG4 antibody targeting PD-1), cemiplimab (a humanized monoclonal IgG4 targeting PD-1), atezolizumab (a humanized IgG1 antibody targeting PD-L1), and durvalumab (a humanized IgG1 antibody target PD-L1). Intriguingly, ICIs showed unexpected long-lasting responses and survival in NSCLC stage IV patients. In a cohort of 129 patients with a minimum follow-up of 58.25 months, 16% of NSCLC stage IV patients showed overall survival with nivolumab treatment [4].

Despite the tremendous success of ICIs in the treatment of NSCLC, approximately 80% of NSCLC patients do not benefit from ICIs due to primary resistance (no initial response) or acquired resistance (tumor relapse after an initial response) [5]. Identifying the underlying mechanisms of ICIs resistance has been an urgent task to improve benefits of immunotherapy against cancer and provides rationales for combinational treatment. In this review, mechanisms related to ICIs resistance are presented. First, primary resistance against ICIs in NSCLC are listed based on the cancer-immunity cycle. Second, acquired resistance induced by FDA-approved ICIs in NSCLC treatment are summarized.

**Figure 1:**
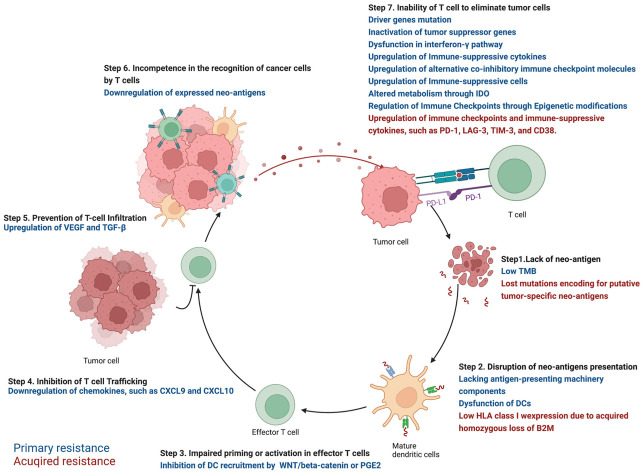
Primary and acquired mechanisms against ICIs in NSCLC.

In the well-known cancer-immunity cycle (shown in Figure 1), tumor cell death mediated by cytotoxic T cell can be divided into 7 steps as follows [6]: antigen presentation cells (APCs), primarily dendritic cells (DCs), capture neo-antigens released from tumor cells (step 1); neo-antigens are presented to the corresponding T cell receptor (TCR) of naïve T cells by major histocompatibility complex (MHC) on APCs (step 2); priming and activation of effector T cells (step 3); trafficking of T cell from lymph nodes to tumor (step 4); infiltration of T cells into tumor (step 5); recognition of cancer cells by T cells (step 6); and killing of tumor cells (step 7). In step 3 and step 7, several co-inhibitory immune checkpoints are expressed in T cells to avoid autoimmunity, such as CTLA-4 and PD-1. CTLA-4 combines with B7 expressed in APCs to terminate T-cell activation through the disruption of co-stimulatory signal from the binding of CD28-B7 pathway [7], while the combination between PD-1 and PD-L1/PD-L2 inhibits T-cell activation and proliferation through promoting apoptosis in effector T cells and inhibiting apoptosis in regulatory T cells (Tregs) [8]. Tumor cells escaping the cancer-immunity cycle disrupt these steps and several ICIs are developed to block inhibition and promote the elimination of cancer cells by activated T cells. However, the dysfunction of each step in the cycle leads to ICI resistance.

## 2. Primary Resistance against ICIs in NSCLC

### 2.1. Step 1. Lack of Neo-Antigen Due to Low TMB

Tumor mutational burden (TMB) is termed as the frequency of non-synonymous somatic mutations per DNA megabase (Mb) in the coding genome of the tumor [9]. Expression of mutated genes produces neo-antigens that can be recognized by the immune system through MHC class I (MHC-I), while the lack of neo-antigens fails to initiate cancer-immunity cycle and leads to ICI resistance. In NSCLC, TMB showed a positive association with tumor neo-antigen load and low TMB is a mechanism of primary resistance against ICIs [10,11]. Patients harboring low TMB showed lower objective response rate to ICI compared with high TMB group [12]. In advanced or recurrent NSCLC patients with low or medium TMB, both nivolumab and nivolumab plus ipilimumab treatment showed a shorter progression-free survival (PFS) compared with chemotherapy (4.1 versus 6.9 months, hazard ratio = 1.82, 95% CI: 1.30–2.55) [13].

### 2.2. Step 2. Disruption of Neo-Antigens Presentation

The disruption of neo-antigen presentation can be caused by the downregulation of antigen-presenting machinery components or the dysfunction of DCs.

Lacking antigen-presenting machinery components is a mechanism for both primary and acquired resistance, such as beta-2-microglobulin (β2M) and human leukocyte antigen (HLA). β2M is a part of HLA I complex and its loss was associated with an unfavorable prognosis in NSCLC [14], while loss of heterozygosity in HLA genes is reported in 40% of NSCLCs, leading to the dysfunction of MHC-I presentation and resistance against ICIs [15]. Interferon (IFN)-γ signaling pathway promoted the expression of transporter associated with antigen processing (TAP) and MHC molecules, while mutations in the pathway dampen antigen presentation and induce primary resistance [16].

The dysfunction of DCs is another mechanism to disrupt antigen presentation. Several factors can affect the differentiation, activation of DCs [17]. Interleukin (IL)-6, vascular endothelial growth factor (VEGF), and granulocyte colony-stimulating factor (G-CSF) directly inhibited the differentiation of CD34^+^ precursor cells into DCs [18]. STING signaling pathway promoted the expression of IFN-β to activate DCs, while STING knockout mice showed a lack of effector T-cell due to insufficient DCs [19]. The gut microbiota also plays a role in the cancer-immunity cycle through the activation of DCs in the intestine. Bifidobacterium-feeding mice showed upregulation of MHC-II in DCs to facilitate antigen presentation [20]. In NSCLC patients, the prior antibiotic administration was associated with a higher likelihood of primary resistance against ICIs and shorter OS (2.5 versus 26 months, *p* < 0.001) [21].

### 2.3. Step 3. Impaired Priming or Activation in Effector T Cells

IFN-α signaling pathway plays an important role in the priming of T cells by DCs and mutations in the pathway inhibits the priming and activation of effector T-cells [22], while the activation of WNT/beta-catenin pathway inhibits T-cell priming through the inhibition of DC recruitment and induces resistance against ICIs [23]. Prostaglandin E2 (PGE2) also inhibited the recruitment of DCs to tumor through decreasing the expression of chemokine receptor [24].

### 2.4. Step 4. Inhibition of T Cell Trafficking

Chemokines, such as C-X-C motif chemokine ligand 9 (CXCL9) and CXCL10, promote the trafficking of effector T cells while downregulation of these genes is a mechanism of primary resistance [25]. For example, histone H3 lysine 27 trimethylation (H3K27me3) mediated by zeste homologue 2 (EZH2) and DNA methylation mediated by DNA methyltransferase 1 (DNMT1) suppressed expression of CXCL9 and CXCL10 to inhibit trafficking. Further, epigenetic modulators alleviated the repression and promoted efficacy of anti-PD-L1 inhibitors [26].

### 2.5. Step 5. Prevention of T-Cell Infiltration

VEGF is a key regulator of angiogenesis in tumors, which inhibits the infiltration of T-cells into tumor microenvironments through downregulation of NF-κB-induced endothelial activation [27]. In NSCLC, the activation of RAS/MAPK and PI3K/AKT pathways and the loss of PTEN promotes the expression of VEGF to inhibit T-cell infiltration and induce primary resistance [28]. The upregulation of transforming growth factor beta (TGF-β) in the fibroblast of the tumor microenvironment (TME) reduced effects of anti-PD-L1 inhibitors by restricting T-cell infiltration [29]. Compared with the wild-type group, lung adenocarcinoma patients harboring KEAP1-mutations showed significantly lower CD8+ T cells and dendritic cells infiltrations [30].

### 2.6. Step 6. Incompetence in the Recognition of Cancer Cells by T Cells

In this step, tumor cells decrease the expression of neo-antigens to avoid T cell recognition, such as downregulation of expressed neo-antigens through promoter hypermethylation of genes expressing neo-antigens [31].

### 2.7. Step 7. Inability of T Cell to Eliminate Tumor Cells

In this step, an immune-suppressive TME characterizing by exhausted T-cells and upregulation of PD-L1 is induced through multiple mechanisms, including mutations in oncogenes and tumor suppressor genes, upregulation of alternative immune checkpoints, dysfunctions in INF-γ pathway, expression of immune-suppressive cytokines and cells, metabolism changes, and epigenetic modifications.

#### 2.7.1. Driver Genes Mutation

Mutations of several pathways in NSCLC upregulate PD-L1 expression to achieve immune escape, including EGFR pathway, ALK pathway, RAS/RAF/MAPK pathway, EMT, MYC, and mTOR.

No genomic aberration of EGFR mutation or ALK rearrangement is a common criterion for the clinical application of ICIs in NSCLC. Several studies showed that upregulation of PD-L1 expression should be the reason for the primary resistance against ICIs in NSCLC harboring EGFR mutation or ALK rearrangement. Activation of mutant EGFR in murine bronchial epithelial cells induced the upregulation of PD-1, PD-L1, and CTLA-4, while EGFR inhibitors inhibited the expression of PD-L1 in NSCLC cell lines. [32] EGFR regulated PD-L1 expression through several downstream targets, including PI3K, MAPK, and signal transducer and activator of transcription 3 (STAT3) [33]. Samples from NSCLC patients also demonstrated a positive relationship between expression of PD-L1 and mutant EGFR [34]. In lung cancer cell lines harboring acquired resistance against EGFR tyrosine kinase inhibitors (TKI), there was a correlation between the downregulation of TKI-induced E-cadherin and decreased PD-L1 expression [35]. Moreover, there is a positive association between the EML4–ALK fusion gene and expression of PD-L1 in NSCLC specimens [36]. Forced expression of EML4–ALK promoted the expression of PD-L1 through PI3K/AKT and RAS/RAF/MAPK pathways, which was attenuated by ALK inhibitor.

Activated RAS stabilized PD-L1 mRNA directly and inhibited tristetraprolin through MAPK activation to promote PD-L1 expression in lung cancer cell lines [37]. Through EGFR pathway and EML4-ALK fusion, activated MAPK also promotes the expression of PD-L1 [38]. In erlotinib-resistant NSCLC cells, the inhibition of MAPK decreased the expression of PD-L1 significantly [39].

In erlotinib-resistant NSCLC cells, the upregulation of PD-L1 was associated with MET amplification, and the inhibition of MET significantly decreased PD-L1 in transcriptional and protein levels [39]. Through a post-transcriptional mechanism avoiding significant increasing of mRNA, activated mTOR promotes the recruitment of PD-L1 transcripts with active polysomes to elevate PD-L1 at protein level. Oncogenic activation of AKT-mTOR pathway also promoted PD-L1 expression and the combinational treatment of mTOR inhibitor and PD-L1 antibody inhibited tumor growth in syngeneic and genetically engineered mouse models of lung cancer [40]. MYC binds directly to the promoter of PD-L1 gene to promote its expression [41] and there is a significant association between the expression of MYC and PD-L1 in NSCLC patients [42].

#### 2.7.2. Inactivation of Tumor Suppressor Genes

Inactivation of tumor suppressor genes caused by mutations induces a TME favoring immune escape through the upregulation of PD-L1. In KRAS-driven NSCLC, inactivation of tumor-suppressor gene STK11/LKB1 was associated with downregulation of PD-L1 and suppressed effector T-cells [43]. In addition, in KRAS-mutant lung adenocarcinoma, STK11/LKB1 inactivation induced immunosuppressive TME and innate resistance to PD-1 inhibitors through downregulation of PD-L1 in tumor cells and effector T-cell exclusion [44]. Furthermore, in KRAS-mutant lung cancers, LKB1 loss resulted in marked silencing of STING expression partly by hyper-activation of DNMT1 and EZH2 activity [45]. Somatic mutations of Kelch-like ECH-associated protein 1 (KEAP1) and TP53 were also associated with high TMB and PD-L1 expression in NSCLC patients [44,46], while metastatic NSCLC patients harboring STK11 or KEAP1 showed poorer outcomes with ICI treatment compared with wild-type groups [44].

#### 2.7.3. Dysfunction in Interferon-γ Pathway

IFNγ released by effector T-cells activates Janus kinase (JAK)–signal transducer and activator of transcription (STAT) pathway to promote PD-L1 expression [47]. In NSCLC patients treated with nivolumab, there is a correlation between high IFN-γ mRNA expression and progression-free survival [48]. There was also a positive association between activating mutations in JAK2/JAK3 and upregulation of PD-L1 expression in a cohort of 932 NSCLC patients [49]. Furthermore, mutations in the pathway cause the insensitivity to IFN-γ and following downregulation of PD-L1, which leads to the resistance against ICIs [50].

#### 2.7.4. Immune-Suppressive Cytokines

The releasing of immune-suppressive cytokines suppresses anti-tumor immune responses, including TGF-β, tumor necrosis factor (TNF-α), and IL-6. In NSCLC patients, TGF-β signaling upregulated PD-1 and PD-L1 to promote T-cell anergy and stimulate the recruitment of Tregs to induce resistance [51]. The expression of TNF-α was also associated with the expression of PD-L1 and T-cell immunoglobulin and mucin-domain containing-3 (TIM-3) after ICIs treatment [52]. IL-6 decreased the levels of PD-L1 and MHC-I to induce immune escape and resistance against ICIs [53].

#### 2.7.5. Upregulation of Alternative Co-Inhibitory Immune Checkpoint Molecules

The upregulation of alternative co-inhibitory immune checkpoints on T cells is another mechanism of ICI resistance in NSCLC, including T-cell immunoglobulin and mucin-domain containing-3 (TIM-3) and Lymphocyte Activating 3 (LAG-3) [54]. TIM-3 induces T-cell exhaustion through the activation of apoptosis pathway. In mouse models and patients of lung adenocarcinoma with anti-PD-1 therapy, an increase of TIM-3 was reported [55]. LAG3 is another negative immune regulator inducing exhausted T-cells and it was correlated with the expression of PD-1/PD-L1 in NSCLC patients [56].

#### 2.7.6. Immune-Suppressive Cells

Several immune cells inhibit the function of effector T-cells to dampen the efficacy of ICIs through direct contact, releasing suppressive molecules (TGF-β and IL-10) and upregulation of immune checkpoints (PD-1, CTLA-4, TIM-3, and LAG-3), including T regulatory cell (Treg), B regulatory cell (Breg), M2 Tumor-associated macrophages (TAMs), and Myeloid-derived-suppressor cells (MDSC) [53]. Through the comparison between different NSCLC subtypes, TAM and Breg played a dominant immunosuppressive role in squamous cell carcinomas and adenocarcinomas, respectively [57].

#### 2.7.7. Altered Metabolism through Indoleamine 2,3-Dioxygenase (IDO)

IDO is a time-limiting enzyme catalyzing the kynurenine (kyn) pathway in tryptophan (trp) metabolism, which also works as an inhibitory immune checkpoint by promoting effector T cell apoptosis through trp shortage [58]. In ICI non-responding NSCLC patients, an upregulation of IDO was observed compared with responding group through mass spectrometry [59]. In 14 NSCLC patients treated with nivolumab, there was a significant association between early progress and higher kyn/trp ratio (indicating higher IDO activity), suggesting the possibility of combinational treatment of nivolumab and IDO inhibitor [58].

#### 2.7.8. Regulation of Immune Checkpoints through Epigenetic Modifications

Epigenetics is the study of heritable phenotype changes without changes of DNA sequence, including DNA methylation, post-translational modification, and non-coding RNA [60]. The expression of immune checkpoint molecule is regulated by several epigenetic mechanisms in NSCLC. Hypomethylation of CTLA-4 and PD-1 in tumor tissues compared with matched controls was reported in NSCLC patients, leading to high expression [61]. In the process of epithelial-mesenchymal transition (EMT), transforming growth factorβ-1 (TGFβ-1) inhibited DNA-methyltransferase 1 (DNMT1), promoting the de-methylation of PD-L1 promoter and up-regulation of PD-L1 [62]. Azacytidine, a DNMT inhibitor, induced the upregulation of PD-L1 in both transcriptional and protein levels in several NSCLC cell lines [63]. As a histone methyltransferase, enhancer of zeste homolog 2 (EZH2) is responsible for mono-, di-, and tri-methylation of lysine residue 27 on histone H3 (H3K27) and showed a positive association with higher PD-L1 in lung adenocarcinomas, suggesting the regulation of immune checkpoints molecules through histone modification [64]. MicroRNAs (miRs) also play a role in the immunotherapy through the regulation of PD-L1. Through the direct binds to 3’ untranslated region, miR-34 suppressed the expression of PD-L1, which promoted tumor-infiltrating lymphocytes (TILs) and reduced both macrophages and Treg in a syngeneic mouse model of NSCLC [65]. MiR-200 inhibited PD-L1 expression in both murine and human lung cancer cell lines [66].

## 3. Acquired Resistance in NSCLC

Several mechanisms are overlapped in primary and acquired resistance. As regards the lack of neo-antigens, tumors lost 7 to 18 mutations encoding for putative tumor-specific neo-antigens through the elimination of specific sub-clones or deletion of chromosomal regions containing truncal alterations showed acquired resistance against nivolumab or nivolumab/ipilimumab [67]. For the disruption of antigen presentation, acquired homozygous loss of beta-2-microglobulin (B2M) was identified in an acquired ICI-resistant lung cancer sample, which decreased the expression of HLA class I and disrupted the antigen-presentation [68].

Upregulation of immune checkpoints and immune-suppressive cytokines are also reported in acquired resistance. In lung cancer patients with acquired resistance against anti-PD-1 therapy, the expression of PD-1 and LAG-3 were upregulated [69]. In a prospective cohort of metastatic NSCLC treated with nivolumab, upregulation of lymphoid cells harboring high TIM-3 and monocytic MDSC expressing galectin-9 (the ligand of TIM-3) induced primary or acquired secondary resistance through reducing the production of IFN-γ secreted by effector T-cells [70]. In two NSCLC patients and two mice models of lung adenocarcinoma with anti-PD-1 treatment, the upregulation of TIM-3 was observed, suggesting another mechanism of acquired resistance against anti-PD-1 therapy [55]. In the acquired resistance of NSCLC tumor models against ICIs, IFN-β and all-trans retinoic acid promoted the expression of CD38, which inhibited effector T-cell function through adenosine receptor signaling [71]. The high expression of transforming growth factor-beta-induced protein (TGFBI) is also associated with resistance against nivolumab. In a NSCLC cohort of 33 patients treated with nivolumab, poorer response was observed in high TGFBI group compared with low group [72]. Intriguingly, in 2 cases of NSCLC patients showing acquired resistance against nivolumab, biopsy reported the transformation to small cell lung cancer, suggesting a novel mechanism of acquired resistance [73].

## 4. Future Direction

Currently, personalized medicine approaches mainly focus on the evolution of mutated oncogenes especially driver genes to assess treatment response. With the increasing attention of immunotherapy represented by PD-1 and PD-L1, there is still a lack of effective treatment for targeting microenvironment. In fact, there is a wealth of laboratory and clinical data confirming that the immune status has an important role in indicating the final treatment outcome [74].

As lung cancer environment status is changing with tumor evolution and response to treatment, molecular and functional new mechanism may help improve the targeting of treatment regimens to individual patient-specific conditions. In fact, there is also a large amount of laboratory and clinical data confirming that immune status is an important predictor of final treatment outcome. Recent studies have also begun to use immunological characteristics of microenvironment as a predictive biomarker for evaluating reaction for immunotherapy. At present, many studies are trying to find effective indicators for the efficacy evaluation of immunotherapy; although there are many advances in animal models and in vitro mechanism experiments, it is still an urgent scientific problem to find a reliable indicator to predict the efficacy of immunotherapy.

The complex microenvironment of the lung provides superior conditions for the development of primary lung cancer and extrapulmonary tumor metastasis and provides target resources to be developed for the development of clinical treatments. Mechanistic insights into the tumor reprogramming microenvironmental landscape in lung cancer and the development of specific inhibitory drugs have ushered in a new era of cancer therapy. Some immunotherapies perform exceptionally well compared to standard chemotherapy and finding predictive biomarkers to better leverage the benefits of these therapies in the clinic remains a formidable challenge. Both PD-L1 and tumor mutational burden are used clinically as predictive biomarkers to select patients with NSCLC for immunotherapy. Since neither of them can predict the outcome of immunotherapy accurately, the characteristics of immune microenvironment and cytokine levels are being studied extensively to explore a more effective biomarker for immunotherapy. The concept of universal therapy is a thing of the past, and we have now entered the era of precision medicine for lung cancer, which is now focusing on individual molecular testing and cancer genome analysis to assist clinicians in determining appropriate treatments. To maximize the therapeutic effect, the current stage should combine genetic information with the tumor microenvironmental landscape to develop rationally designed combination therapies. More importantly, the success of targeting angiogenesis and immune cells has rekindled enthusiasm for elucidating the role of other components of the lung tumor environment for clinical assessment in lung cancer prognosis and pathophysiology.
